# Dexmedetomidine modulates neuronal activity of horizontal limbs of diagonal band via α2 adrenergic receptor in mice

**DOI:** 10.1186/s12871-023-02278-8

**Published:** 2023-10-02

**Authors:** Xia-wei Zhang, Lei Chen, Chang-feng Chen, Juan Cheng, Ping-ping Zhang, Lie-cheng Wang

**Affiliations:** 1 Department of Physiology, School of Basic Medical Sciences, Anhui Medical University, 230032 Hefei, China; 2 Departments of Pharmacy, The First Affiliated Hospital of Anhui University of Chinese Medicine, 230031 Hefei, China

**Keywords:** Dexmedetomidine, Basal forebrain, Horizontal limbs of diagonal bundle, α_2_ adrenergic receptor

## Abstract

**Background and objectives:**

Dexmedetomidine (DEX) is widely used in clinical sedation which has little effect on cardiopulmonary inhibition, however the mechanism remains to be elucidated. The basal forebrain (BF) is a key nucleus that controls sleep-wake cycle. The horizontal limbs of diagonal bundle (HDB) is one subregions of the BF. The purpose of this study was to examine whether the possible mechanism of DEX is through the α2 adrenergic receptor of BF (HDB).

**Methods:**

In this study, we investigated the effects of DEX on the BF (HDB) by using whole cell patch clamp recordings. The threshold stimulus intensity, the inter-spike-intervals (ISIs) and the frequency of action potential firing in the BF (HDB) neurons were recorded by application of DEX (2 µM) and co-application of a α_2_ adrenergic receptor antagonist phentolamine (PHEN) (10 µM).

**Results:**

DEX (2 µM) increased the threshold stimulus intensity, inhibited the frequency of action potential firing and enlarged the inter-spike-interval (ISI) in the BF (HDB) neurons. These effects were reversed by co-application of PHEN (10 µM).

**Conclusion:**

Taken together, our findings revealed DEX decreased the discharge activity of BF (HDB) neuron via α_2_ adrenergic receptors.

## Introduction

General anesthetic dexmedetomidine (DEX) is one of the most widely used for sedation and analgesia in intensive care because of its good tolerance and few adverse reactions [1, 2]. Since it produces similar natural sleep effects, most researchers have speculated that it may work through sleep-wake nuclei or related projections [3, 4]. Studies confirmed that it produces sedative or hypnotic effects mainly through the locus coeruleus (LC) which mediates arousal [5, 6]. Because of the electroencephalogram (EEG) produced by DEX similarity to the non-rapid eye movement (NREM) sleep, the idea that an endogenous NREM sleep-promoting system to exert its sedative effects has been proposed [3]. Bilateral ventrolateral preoptic (VLPO) lesions, one of the nuclei regulating NREM sleep, can attenuate the sedative effect of DEX via gamma-aminobutyric (GABA) acid receptor and a α_2_ adrenergic receptor in rats [7]. However, the role of DEX in regulating other nuclei of NREM sleep remains to be elucidated.

The involvement of hypothalamic sleep pathway plays an important role in general anesthesia [8, 9]. The basal forebrain (BF) is a key nucleus that regulates NREM sleep in hypothalamus [10, 11]. There are four main types of neurons in BF, including cholinergic neuron, glutamatergic neuron, parvalbumin positive GABAergic (GABA^PV+^) neuron and somatostatin positive GABAergic (GABA^SOM+^) neuron [12–14]. Among the four types of neurons in the BF nucleus, only GABA^SOM+^ neuron has the effect of promoting NREM sleep [15, 16]. There are some co-expressed receptors in BF, such as adenosine receptor related to homeostasis and 5-hydroxytryptamine (5-HT) receptor related to sleep-wake [17, 18].

DEX, a selective α2 adrenergic receptor agonist, whose sedative effects may occur through interactions with sleep-related neurons in the hypothalamus [19]. GABAergic neurons of BF innervate neocortex inhibitory interneurons in sleep-wake regulation [20]. GABAergic neurons co-expressed α2 adrenergic receptor in BF express c-Fos during sleep [21]. Immunohistochemical evidence suggests that cholinergic and GABAergic neuron distributed in the horizontal limbs of diagonal bundle (HDB) subarea of BF, which associated with sleep-wake regulation [22, 23]. These evidences suggest that DEX may have an effect on neuronal activity of HDB nuclei, but direct evidence is still lacking. Therefore, in this study, we hypothesize that DEX may have an effect on the neuronal activity of the BF (HDB) nucleus, and through α2 adrenergic receptors.

## Materials and methods

Drugs were diluted with extracellular bathing solution to a final concentration of DEX (2 µM, Orion Pharma and Abott, USA) and α_2_ adrenergic receptor antagonist phentolamine (PHEN) (10 µM, Wedily, Hubei) immediately before the experiments [24]. All other drugs (Sigma-Aldrich, St. Louis, USA) and all solutions (Sigma-Aldrich, St. Louis, USA) were applied using a perfusion pump system (Longerpump, Baoding, China).

### Animals

Electrophysiological experiments on brain slices and immunofluorescent staining experiments were performed in male C57BL/6 mice (4 weeks old). Animals were housed under 22–24℃, 40 − 60% humidity with a 12 h:12 h light/dark cycle (lights on at 8:00 am, lights off at 20:00 pm). Food and water were available ad libitum. All experiments were approved by the Experimental Animal Ethics Committee of Anhui Medical University, and adhered to the guidelines of the Institutional Animal Care Unit Committee of Anhui Medical University. All methods were carried out in accordance with relevant guidelines and regulations.

### Brain slices preparation

Mice were anesthetized with isoflurane and their brains were quickly decapitated. The slices (300 μm) were cut using a vibratome (Leica Biosystems Inc. Buffalo Grove, United States) which using the prepared continuous in oxygenated (95% O_2_/5% CO_2_) artificial cerebrospinal fluid (ACSF) containing (mM) 60 NaCl, 1.25 KCl, 25 NaHCO_3_, 1.25 NaH_2_PO_4_, 25 D-Glucose, 120 Sucrose, 0.1 CaCl_2_, 3 MgCl_2_, 5 sodium pyruvate, 2.5 ascorbic acid, pH (7.4 ± 0.5), at 4 °C. Then, the slices were removed and placed into oxygenated ACSF containing (mM) 125 NaCl, 1.25 KCl, 1.25 NaH_2_PO_4_, 25 NaHCO_3_, 25 D-Gluose, 5 sodium pyruvate, 2.5 ascorbic acid, pH (7.4 ± 0.5), at 25 °C for 1 h. The slices were transferred to a submersion chamber perfused with the oxygenated ACSF (30 °C) with a constant perfusion rate at 3ml/min for whole-cell patch clamp recordings.

### Whole-cell patch clamp recordings

The neurons were recorded using a MultiClamp-700B amplifier (Molecular Device, San Jose, CA, USA) in current-clamp mode. Electrical signals were recorded using the pClamp-10 software (Axon Instrument Inc., San Jose, CA, USA) for data acquisition and analysis. The pipette solution for recording the spikes contained (in mM) 135 K-gluconate, 10 KCl, 10 HEPES, 0.1 EGTA, 5 Mg-ATP, 0.5 Na-GTP, pH (7.4 ± 0.1). The pipette solutions were freshly made and filtered (0.22 μm) before use. The osmolarity was 305–310 mOsmol, and the pipette resistance was 4–6 MΩ.

BF (HDB) neuron excitability activity was assessed by the threshold stimulus intensity, the inter-spike intervals (ISIs) and the firing frequency of action potentials (APs). The threshold stimulus intensity and ISIs were induced by injecting depolarization pulses under the current mode. The neural-intrinsic properties of the threshold stimulus intensity were used to evaluate the neuronal excitability, which were obtained by injecting a current of threshold stimulus pulses that just enough generated the APs. The ISIs, which represented the time interval between two neighboring spikes under the same stimulus intensity and duration was evaluated the ability to convert excitatory inputs into digital spikes. The firing frequency of spontaneous APs recorded in gap-free mode represents the state of neurons under normal physiological conditions without giving any stimulation. We recorded threshold stimulus intensity, ISIs and frequency before the drug application (approximately 10 min). The three indexes were recorded again during and after the drug application.

### Histology and immunohistochemistry

Mice were deeply anesthetized by isoflurane and transcardially perfused with saline (0.9%) followed by 4% paraformaldehyde in PBS. After removal, brains stayed in 4% paraformaldehyde overnight. For cryoprotection, brains were stored in sequence in 20% and 30% sucrose (w/v) in PBS solution at least 1 night. Brains were sliced in 40 μm sections using a frozen slicing machine (CM3050S, Leica). For immunohistochemistry, membrane permeability were increased by using triton-100 (0.03%) and binding sites were blocked by incubating the brain sections in 5% bovine serum albumin (BSA). Brain sections were incubated with the rabbit polyclonal antibody anti-ADRA2 (BIOSS, 1:200, 1062R) diluted in blocking solution overnight. A species-specific secondary antibody anti-Rabbit IgG (H + L) 488 (Thermo A-21,206, 1:800) was diluted in PBS and applied for 2 h at room temperature. Fluorescence images were taken using a confocal microscope (LSM 880 + airyscan, Zeiss).

### Statistical analyses

The data from the electrophysiological recordings are presented as the mean ± standard error of the mean (SEM). Statistical analysis was performed using Prism 7.0 (GraphPad Software). The software that analyzes is Clamfit 11. Repeated-measures one-way ANOVA with post hoc comparisons was used in the statistical comparisons of the experimental data before, during and after drug administration in the threshold stimulus intensity, ISIs and firing frequency.

## Results

### DEX decreased the activity of BF (HDB) nucleus neurons

**Fig. 1:**
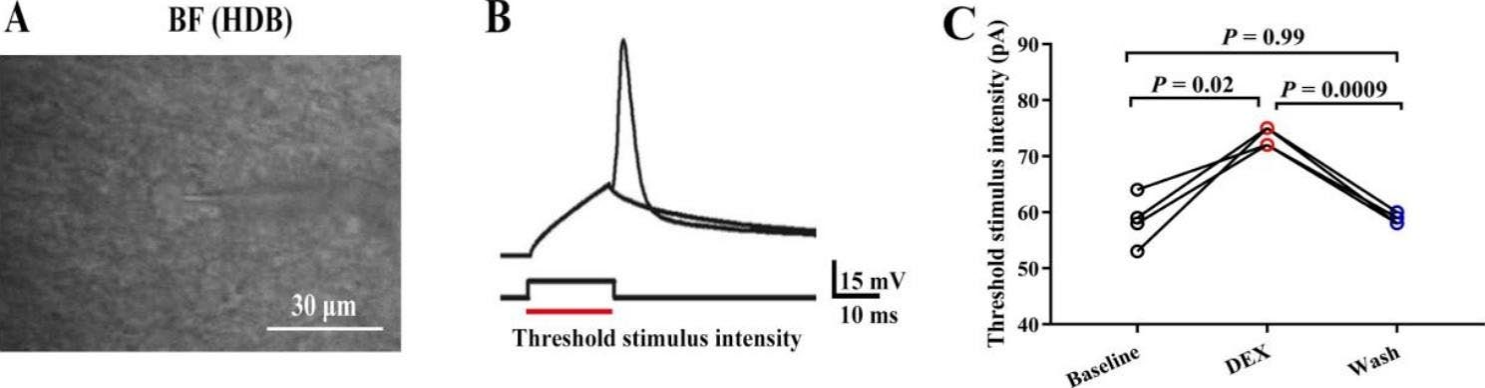
Threshold stimulus intensity in the neurons of the BF (HDB) was increased after DEX administration. (**A**) The neurons of whole-cell recording and electrodes under a microscope. (**B**) Diagram describing the intensity of the threshold stimulus under current mode. (**C**) Statistical data of threshold stimulus intensity in the neurons of the BF (HDB) after DEX administration (*P* < 0.05, one-way ANOVA followed by Tukey’s test, Baseline vs. DEX, DEX vs. Wash, n = 4 neurons from 4 mice)

**Fig. 2:**
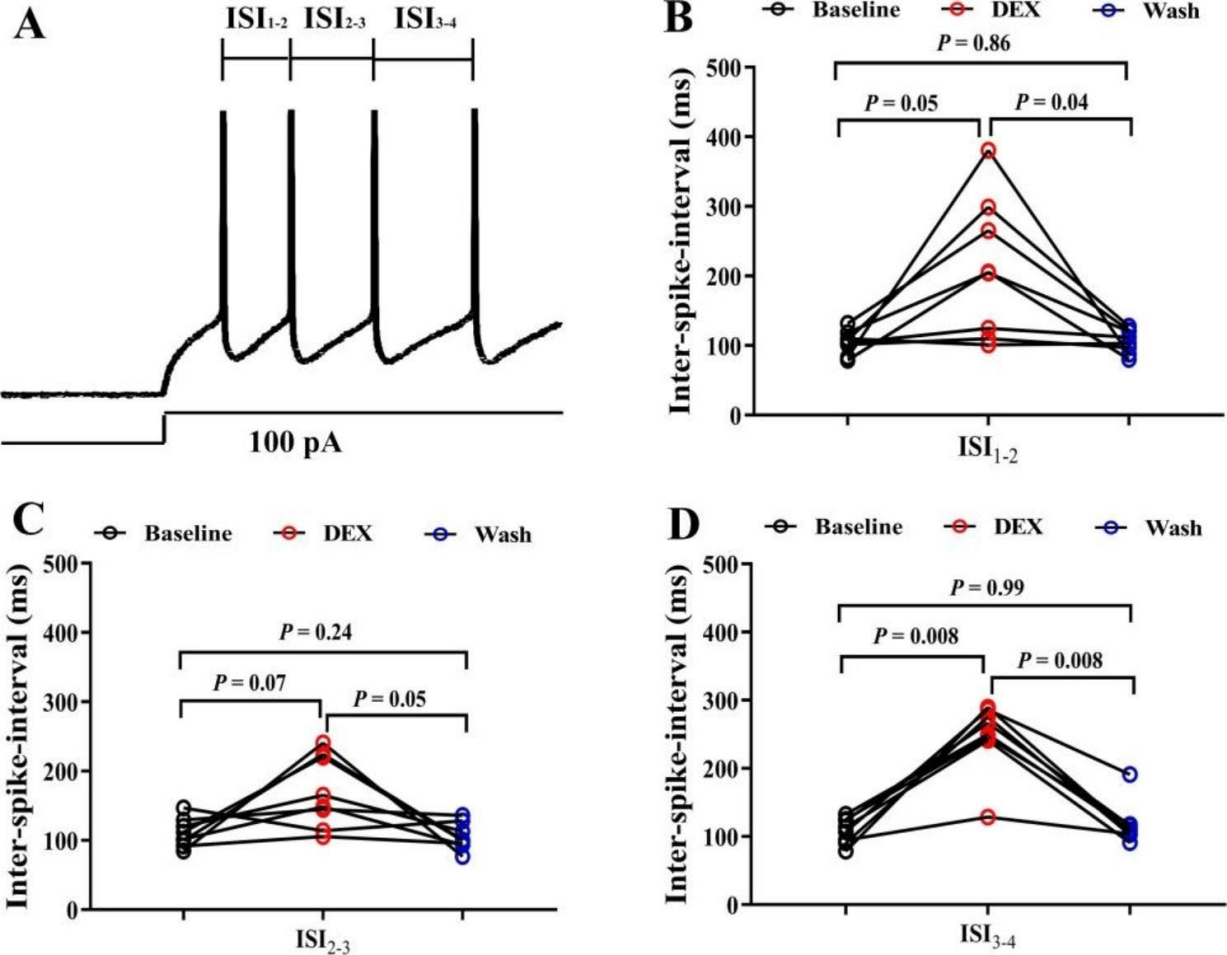
ISIs in the neurons of the BF (HDB) was enlarged after DEX administration. (**A**) Diagram describing the ISIs of the evoked APs under the same intensity stimulus. (**B**)-(**D**) Statistical data of ISIs(ISI_1 − 2_, ISI_2 − 3_, ISI_3 − 4_) in the neurons of the BF (HDB) before, during and after DEX administration(one-way ANOVA followed by Tukey’s test, Baseline vs. DEX, DEX vs. Wash, n = 8 neurons from 8 mice)

The specific patterns generated by injection current can characterize the intrinsic characteristics of neurons. We recorded the threshold stimulus intensity and ISIs to detect the excitability of neurons in BF (HDB). The intensity of the injected depolarization pulse that just induced an APs was called the threshold stimulus intensity. Results showed that the threshold stimulus intensity was increased significantly after administration DEX (Fig. 1A-C, P < 0.05, one-way ANOVA followed by Tukey’s test, Baseline vs. DEX, n = 4 neurons from 4 mice). After perfusing the ASCF, the threshold stimulus intensity recovered to the baseline level (Fig. 1C, P < 0.05, one-way ANOVA followed by Tukey’s test, DEX vs. Wash, n = 4 neurons from 4 mice). Then, the ISIs, which represented the time interval between two neighboring spikes under the same stimulus intensity and duration, were also measured (Fig. 2A). DEX significantly enlarged ISIs (Fig. 2B-D, P < 0.05, one-way ANOVA followed by Tukey’s test, Baseline vs. DEX, n = 8 neurons from 8 mice). At the end of the experiments, ISIs recovered to the baseline level (Fig. 2B-D, P < 0.05, one-way ANOVA followed by Tukey’s test, DEX vs. Wash, n = 8 neurons from 8 mice).

**Fig. 3:**
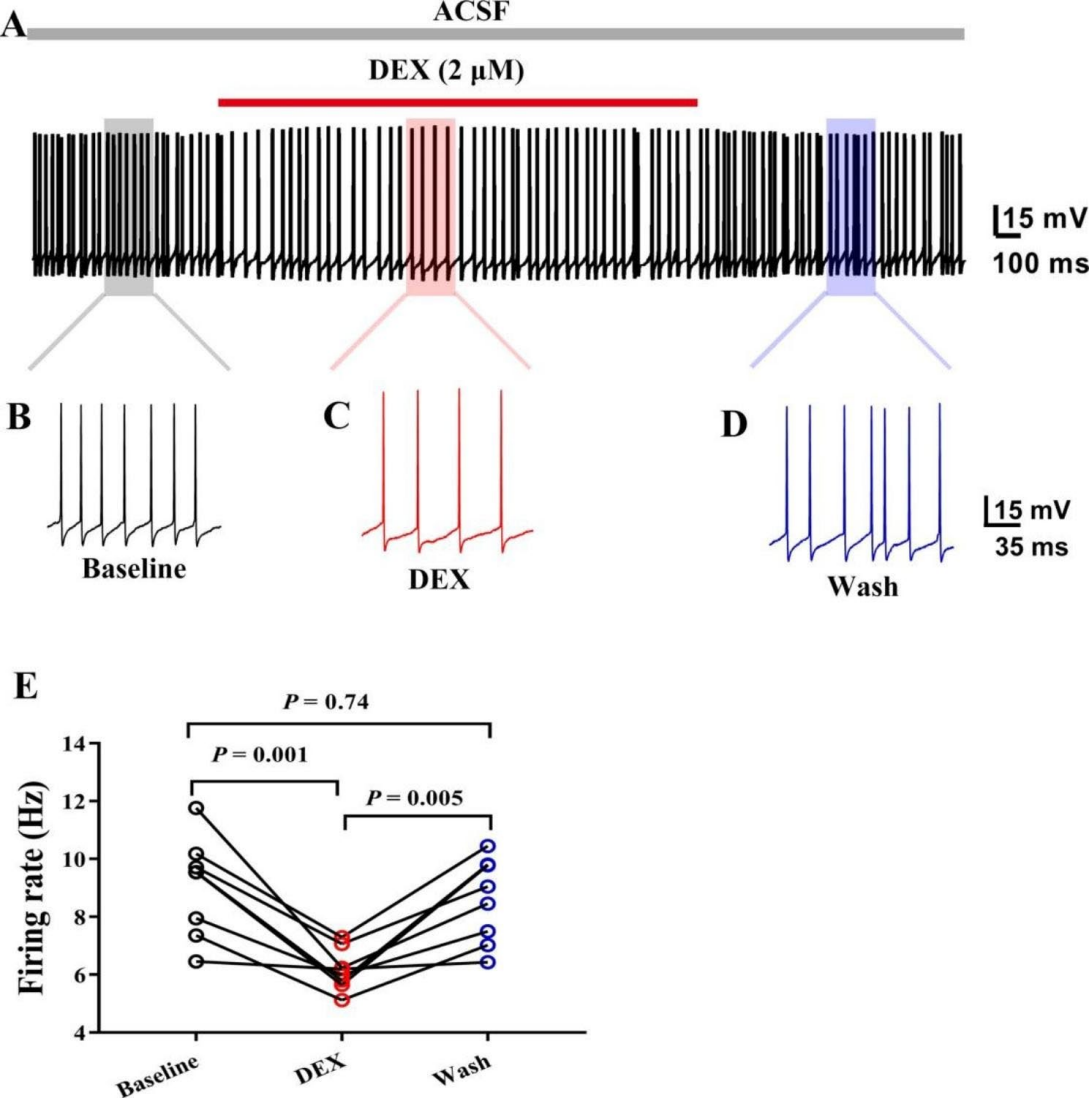
The spontaneous action potentials was inhibited by DEX administration. (**A**) Schematic diagram of action potential before, during and after DEX administration. The gray, red and blue rectangles have the same length of time. (**B**-**D**) are enlarged tracess of the corresponding position in (**A**) represented the baseline, DEX, and Wash-out respectively. (**E**) Statistical data for the frequency of spontaneous action potentials before, during and after DEX administration (one-way ANOVA followed by Tukey’s test, Baseline vs. DEX, DEX vs. Wash, n = 8 neurons from 8 mice)

Similarly, spontaneous firing frequency of APs were recorded in current gap-free mode. Spontaneous discharge traces before, during after DEX were recorded (Fig. 3A-D). DEX significantly decreased the firing frequency (Fig. 3E, P < 0.05, one-way ANOVA followed by Tukey’s test, Baseline vs. DEX, n = 8 neurons from 8 mice). At the end of the experiments, the firing frequency recovered to the baseline level (Fig. 3E, P < 0.05, one-way ANOVA followed by Tukey’s test, DEX vs. Wash, n = 8 neurons from 8 mice).

### Dex modulates neuronal activity of BF (HDB) via α2 adrenergic receptor

**Fig. 4:**
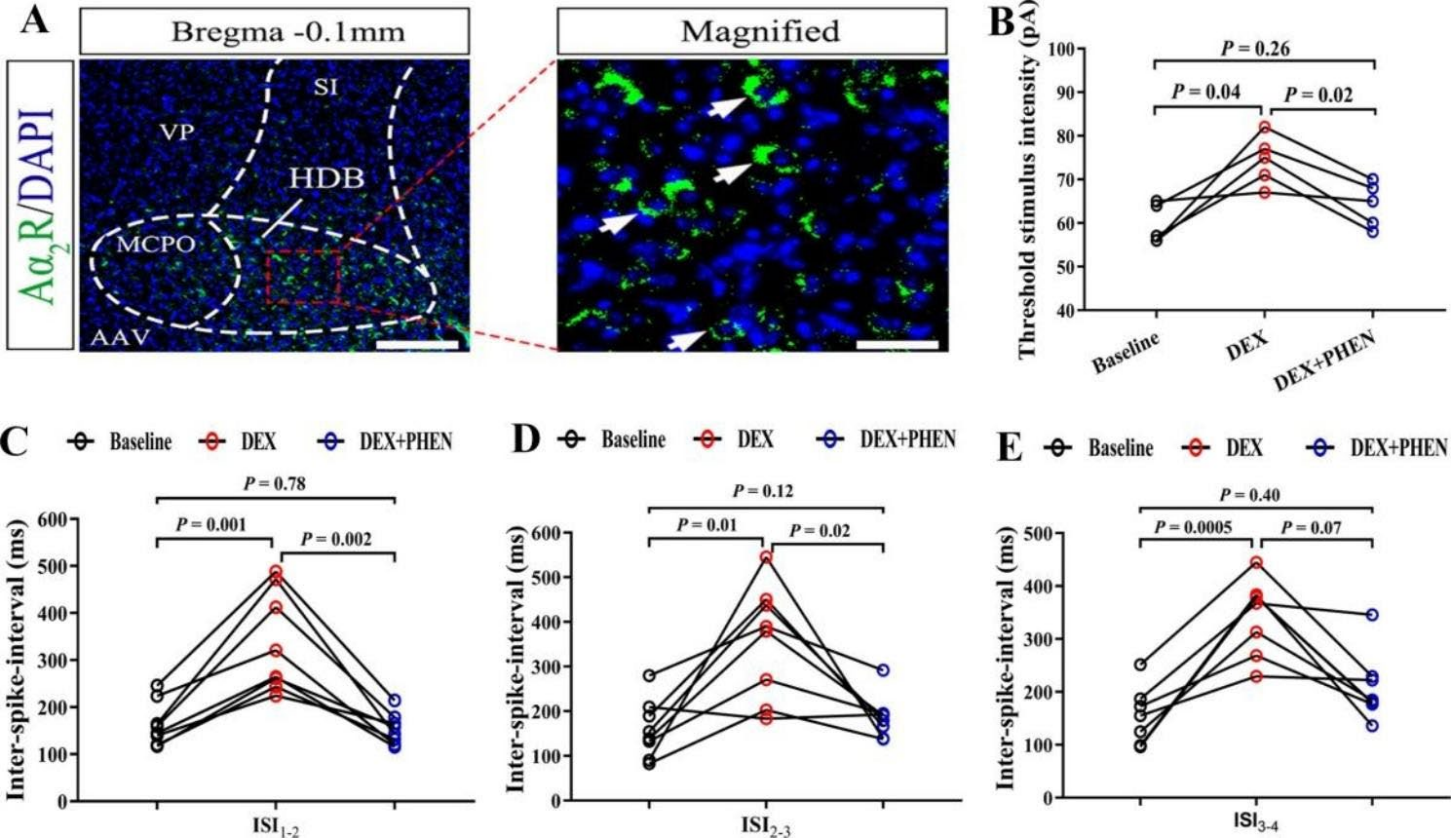
The change of threshold stimulus intensity and ISIs in the neurons of the BF (HDB) caused by DEX was eliminated by PHEN. (**A**) is the staining diagram of α2 adrenergic receptor in the BF(HDB). Blue and green represented DAPI and α2 adrenergic receptor. Scale bar: 200 μm (Left), 20 μm (Right). (**B**) Statistical data of threshold stimulus intensity in the neurons of the BF (HDB) after DEX and DEX + PHEN administration (one-way ANOVA followed by Tukey’s test,, Baseline vs. DEX, DEX vs. DEX + PHEN, n = 5 neurons from 5 mice). (**C**)-(**E**) Statistical data of ISIs (ISI_1 − 2_, ISI_2 − 3_, ISI_3 − 4_) in the neurons of the BF (HDB) before, during and after DEX administration (one-way ANOVA followed by Tukey’s test, Baseline vs. DEX, DEX vs. Wash, n = 8 neurons from 8 mice)

**Fig. 5:**
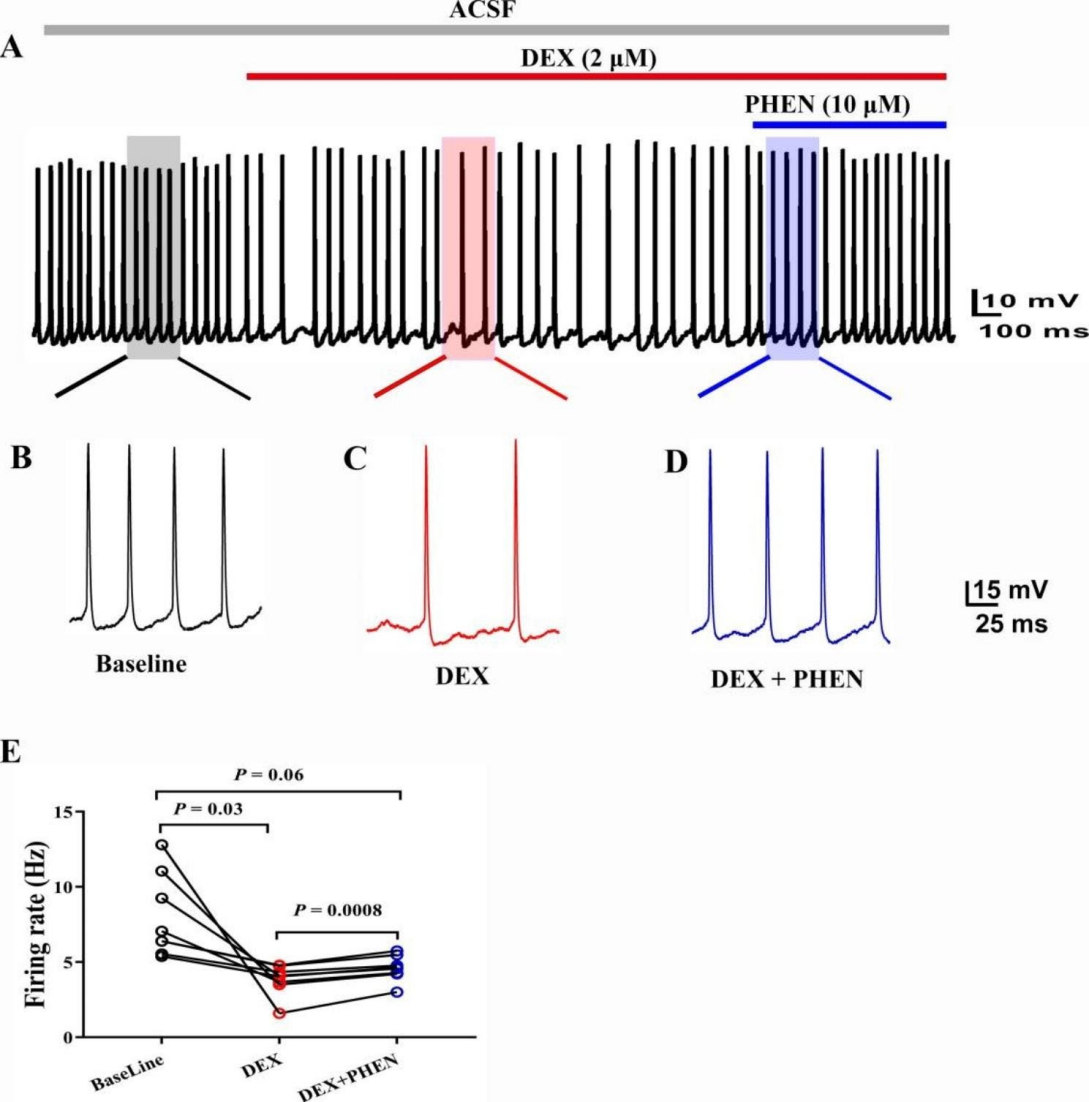
The change of spontaneous action potentials caused by DEX was eliminated by PHEN. (**A**) Schematic diagram of action potential before, during DEX administration and after DEX + PHEN administration. The gray, red and blue rectangles have the same length of time. (**B**-**D**) are enlarged tracess of the corresponding position in (**A**) represented the baseline, DEX, and Wash-out respectively. (**E**) Statistical data for the frequency of spontaneous action potentials before, during DEX administration and after DEX + PHEN administration (one-way ANOVA followed by Tukey’s test, Baseline vs. DEX, DEX vs. DEX + PHEN, n = 8 neurons from 8 mice)

In order to understand the mechanism of the effect of DEX on BF (HDB), we first conducted immunofluorescence staining experiment. Given that DEX is a α2 adrenergic receptor agonist, we observe the distribution of α2 adrenergic receptors in the BF (HDB) first. The results showed that there were a large number of α2 adrenergic receptors in BF (HDB) (Fig. 4A). Therefore, we detected whether could reverse this inhibitory effect by administration a α2 adrenergic receptors antagonists PHEN. We found the effects of threshold stimulus intensity (Fig. 4B, P < 0.05, one-way ANOVA followed by Tukey’s test, Baseline vs. DEX, DEX vs. DEX + PHEN, n = 5 neurons from 5 mice), ISI (Fig. 4C-E, P < 0.05, one-way ANOVA followed by Tukey’s test, Baseline vs. DEX, DEX vs. DEX + PHEN, n = 8 neurons from 8 mice) and firing frequency (Fig. 5A-E, P < 0.05, one-way ANOVA followed by Tukey’s test, Baseline vs. DEX, DEX vs. DEX + PHEN, n = 8 neurons from 8 mice) of APs above by administration DEX were reversed after perfusing DEX + PHEN.

## Discussion

In this study, we confirmed that in the presence of DEX, the threshold stimulation intensity of the evoked APs in BF (HDB) neurons increased, ISIs of the evoked APs enlarged and the frequency of spontaneous APs decreased. Furthermore, we found that α2 adrenergic receptor antagonists PHEN could reverse the effects of DEX on neurons in the BF (HDB). This founding may add a certain theoretical basis for the use of anesthetics in clinic.

### DEX decreased the activity of BF (HDB) nucleus neurons

It was suggested that DEX can reduce neuronal activity of BF (HDB) by comparing the threshold stimulation intensity, ISIs of the evoked APs, and the firing frequency of spontaneous APs (Figs. 1, 2 and 3). This is consistent with previous studies showing that other anesthetics have an inhibitory effect on neurons of the sleep-wake nucleus in the brain. For example, propofol can reduce the intrinsic excitability of cholinergic neurons in substantia innominata (SI) subregion of BF nucleus [25]. There is also evidence that propofol significantly decreased c-Fos expression in wake-related systems [26]. Arousal-related nuclei tuberomammillary nucleus (TMN) histaminergic neurons might be a potential mediator of general anesthetic actions [27]. Activation of serotonergic neurons in the dorsal raphe nucleus (DRN) could facilitate emergence from general anesthesia [28]. Isoflurane and halothane have effect on sleep-active neurons in VLPO [29]. Sleep-promoting preoptic area (POA) neurons are activated by various anesthetics [30]. Zhang et al. found neurons in the mouse lateral preoptic hypothalamic area were sufficient for DEX-induced sedation, as these neurons became active during dex-induced sedation and reactivating them using c-fos activity tagging produced NREM-like sedation [31]. Furthermore, within the lateral preoptic area, genetically lesioning galanin neurons reduced DEX’s ability to induce sedation [32]. Due to the limitation of technology, the research of anesthetics on the central brain is usually limited to a few brain areas or a specific neural circuit. However, whether the inhibitory effect of anesthetics on neurons in the brain is limited or widespread remains unclear. In the future, this is still a problem worth paying attention to.

### Dex modulates neuronal activity of BF (HDB) via α2 adrenergic receptor

After administration of antagonist PHEN, the effect of DXE on neuronal activity of BF (HDB) was eliminated (Figs. 4 and 5). The results of immunofluorescence showed that there were a large number of α2 adrenergic receptors in the BF (HDB) (Fig. 4), this is consistent with the above results. However, the types of neurons affected in our study remain unclear. Previous studies focused more on cholinergic neurons in BF (HDB), which are normally arousal-active neurons [33, 34]. Previous studies demostrated alterations of cholinergic neurons in BF (HDB) may give rise to cognitive alterations in neuropsychiatric disorders, Parkinson’s disease and Alzheimer’s disease [35–37]. As we all know, The α2 adrenergic receptors is both excitatory and inhibitory. It can be excitatory and couple via Gs [38]. For example, in the dorsal bed nucleus of the stria terminalis, alpha2 agonists can direct cause excitation of target neurons [39]. In our study, Dex activated α2 adrenergic receptor of BF (HDB) neurons has an inhibitory effect, suggests that the neurons affected here may be on inhibitory GABAergic neurons. The action potential firing pattern we recorded was consistent with the fast-firing neuron which is GABAergic of BF (HDB) in previous studies [40, 41]. Studies shown that HDB contain distinct populations of GABAergic neuron, meanwhile it can interact with cholinergic neurons [42–44]. There are two main types of GABAergic neurons in the BF (HDB), GABA^PV+^ and GABA^SOM+^. PV-labelled neurons was be predominately located in vertical limb of the diagonal band (VDB), and much lower in BF (HDB) [45]. We speculate that the neurons recorded in our experiment may be GABA^SOM+^ neurons, but this needs to be further verified in the future.

## Conclusion

In the present study, Dex modulates neuronal activity of BF (HDB) via α2 adrenergic Receptor in mice were demonstrated. The key findings of this study are: (1) DEX have an inhibitory effect on the neuronal activity of the BF (HDB) nucleus. (2) Dex modulates neuronal activity of BF (HDB) partly via α2 adrenergic receptor.

## Data Availability

All materials used for the preparation of this manuscript are publicly available. The datasets generated available from the corresponding author upon reasonable request.

## Abbreviations

DEX: dexmedetomidine
LC: locus coeruleus
EEG: electroencephalogram
NREM: non-rapid eye movement
VLPO: ventrolateral preoptic
GABA: gamma-aminobutyric
α2A: α_2_ adrenergic
BF: basal forebrain
GABA^PV+^: parvalbumin positive GABAergic
GABA^SOM+^: somatostatin positive GABAergic
5-HT: 5-hydroxytryptamine
HDB: horizontal limbs of diagonal band
PHEN: phentolamine
ACSF: artifificial cerebrospinal fluid
ISIs: inter-spike intervals
APs: action potentials
BSA: bovine serum albumin
SEM: standard error of the mean
SI: substantia innominata
TMN: tuberomammillary nucleus
DRN: dorsal raphe nucleus
POA: preoptic area
VDB: vertical limb of the diagonal band

## References

[1] Eizaga Rebollar R, García Palacios MV, Fernández Riobó MC, Torres Morera LM (2022). Dexmedetomidine and perioperative analgesia in children. Rev Esp Anestesiol Reanim (Engl Ed.

[2] Tasbihgou SR, Barends CRM, Absalom AR (2022). The role of dexmedetomidine in neurosurgery. Best Pract Res Clin Anaesthesiol.

[3] Moody OA, Zhang ER, Vincent KF, Kato R, Melonakos ED, Nehs CJ, Solt K (2021). The neural circuits underlying General Anesthesia and Sleep. Anesth Analg.

[4] Torao-Angosto M, Manasanch A, Mattia M, Sanchez-Vives MV (2021). Up and Down States during slow oscillations in Slow-Wave Sleep and different levels of Anesthesia. Front Syst Neurosci.

[5] Correa-Sales C, Rabin BC, Maze M (1992). A hypnotic response to dexmedetomidine, an alpha 2 agonist, is mediated in the locus coeruleus in rats. Anesthesiology.

[6] Guo TZ, Jiang JY, Buttermann AE, Maze M (1996). Dexmedetomidine injection into the locus ceruleus produces antinociception. Anesthesiology.

[7] Nelson LE, Lu J, Guo T, Saper CB, Franks NP, Maze M (2003). The alpha2-adrenoceptor agonist dexmedetomidine converges on an endogenous sleep-promoting pathway to exert its sedative effects. Anesthesiology.

[8] Zecharia AY, Nelson LE, Gent TC, Schumacher M, Jurd R, Rudolph U, Brickley SG, Maze M, Franks NP (2009). The involvement of hypothalamic sleep pathways in general anesthesia: testing the hypothesis using the GABAA receptor beta3N265M knock-in mouse. J Neurosci.

[9] Zhong H, Tong L, Gu N, Gao F, Lu Y, Xie RG, Liu J, Li X, Bergeron R, Pomeranz LE, Mackie K, Wang F, Luo CX, Ren Y, Wu SX, Xie Z, Xu L, Li J, Dong H, Xiong L, Zhang X (2017). Endocannabinoid signaling in hypothalamic circuits regulates arousal from general anesthesia in mice. J Clin Invest.

[10] Wu Y, Wang L, Yang F, Xi W (2020). Neural circuits for sleep-wake regulation. Adv Exp Med Biol.

[11] Xu M, Chung S, Zhang S, Zhong P, Ma C, Chang WC, Weissbourd B, Sakai N, Luo L, Nishino S, Dan Y (2015). Basal forebrain circuit for sleep-wake control. Nat Neurosci.

[12] Do JP, Xu M, Lee SH, Chang WC, Zhang S, Chung S, Yung TJ, Fan JL, Miyamichi K, Luo L, Dan Y (2016). Cell type-specific long-range connections of basal forebrain circuit. Elife.

[13] Szymusiak R (1995). Magnocellular nuclei of the basal forebrain: substrates of sleep and arousal regulation. Sleep.

[14] Zaborszky L, Duque A (2003). Sleep-wake mechanisms and basal forebrain circuitry. Front Biosci.

[15] McKenna JT, Thankachan S, Uygun DS, Shukla C, McNally JM, Schiffino FL, Cordeira J, Katsuki F, Zant JC, Gamble MC, Deisseroth K, McCarley RW, Brown RE, Strecker RE, Basheer R (2020). Basal forebrain parvalbumin neurons mediate arousals from Sleep Induced by Hypercarbia or Auditory Stimuli. Curr Biol.

[16] Zant JC, Kim T, Prokai L, Szarka S, McNally J, McKenna JT, Shukla C, Yang C, Kalinchuk AV, McCarley RW, Brown RE, Basheer R (2016). Cholinergic neurons in the basal forebrain promote wakefulness by actions on neighboring non-cholinergic neurons: an opto-Dialysis study. J Neurosci.

[17] Monti JM, Jantos H (2014). The role of serotonin 5-HT7 receptor in regulating sleep and wakefulness. Rev Neurosci.

[18] Peng W, Wu Z, Song K, Zhang S, Li Y, Xu M (2020). Regulation of sleep homeostasis mediator adenosine by basal forebrain glutamatergic neurons. Science.

[19] Yu X, Franks NP, Wisden W (2018). Sleep and Sedative States Induced by targeting the histamine and Noradrenergic Systems. Front Neural Circuits.

[20] Freund TF, Meskenaite V (1992). Gamma-Aminobutyric acid-containing basal forebrain neurons innervate inhibitory interneurons in the neocortex. Proc Natl Acad Sci U S A.

[21] Modirrousta M, Mainville L, Jones BE (2004). Gabaergic neurons with alpha2-adrenergic receptors in basal forebrain and preoptic area express c-Fos during sleep. Neuroscience.

[22] Brashear HR, Zaborszky L, Heimer L (1986). Distribution of GABAergic and cholinergic neurons in the rat diagonal band. Neuroscience.

[23] Lüttgen M, Ogren SO, Meister B (2005). 5-HT1A receptor mRNA and immunoreactivity in the rat medial septum/diagonal band of Broca-relationships to GABAergic and cholinergic neurons. J Chem Neuroanat.

[24] Qiu G, Wu Y, Yang Z, Li L, Zhu X, Wang Y, Sun W, Dong H, Li Y, Hu J (2020). Dexmedetomidine activation of dopamine neurons in the ventral Tegmental Area attenuates the depth of Sedation in mice. Anesthesiology.

[25] Chen L, Yang ZL, Cheng J, Zhang PP, Zhang LS, Liu XS, Wang LC (2019). Propofol decreases the excitability of cholinergic neurons in mouse basal forebrain via GABAA receptors. Acta Pharmacol Sin.

[26] Yue XF, Wang AZ, Hou YP, Fan K (2021). Effects of propofol on sleep architecture and sleep-wake systems in rats. Behav Brain Res.

[27] Luo T, Leung LS (2011). Involvement of tuberomamillary histaminergic neurons in isoflurane anesthesia. Anesthesiology.

[28] Li A, Li R, Ouyang P, Li H, Wang S, Zhang X, Wang D, Ran M, Zhao G, Yang Q, Zhu Z, Dong H, Zhang H (2021). Dorsal raphe serotonergic neurons promote arousal from isoflurane anesthesia. CNS Neurosci Ther.

[29] Moore JT, Chen J, Han B, Meng QC, Veasey SC, Beck SG, Kelz MB (2021). Direct activation of sleep-promoting VLPO neurons by volatile anesthetics contributes to anesthetic hypnosis. Curr Biol.

[30] Reitz SL, Kelz MB (2021). Preoptic area modulation of Arousal in Natural and Drug Induced Unconscious States. Front Neurosci.

[31] Zhang Z, Ferretti V, Güntan İ, Moro A, Steinberg EA, Ye Z, Zecharia AY, Yu X, Vyssotski AL, Brickley SG, Yustos R, Pillidge ZE, Harding EC, Wisden W, Franks NP (2015). Neuronal ensembles sufficient for recovery sleep and the sedative actions of α2 adrenergic agonists. Nat Neurosci.

[32] Ma Y, Miracca G, Yu X, Harding EC, Miao A, Yustos R, Vyssotski AL, Franks NP, Wisden W (2019). Galanin neurons Unite Sleep Homeostasis and α2-Adrenergic sedation. Curr Biol.

[33] Irmak SO, de Lecea L (2014). Basal forebrain cholinergic modulation of sleep transitions. Sleep.

[34] Luo TY, Cai S, Qin ZX, Yang SC, Shu Y, Liu CX, Zhang Y, Zhang L, Zhou L, Yu T, Yu SY (2020). Basal forebrain cholinergic activity modulates isoflurane and Propofol Anesthesia. Front Neurosci.

[35] Liu AKL, Gentleman SM (2020). The diagonal band of Broca in health and disease. Handb Clin Neurol.

[36] Okada K, Nishizawa K, Kobayashi T, Sakata S, Hashimoto K, Kobayashi K (2021). Different cholinergic cell groups in the basal forebrain regulate social interaction and social recognition memory. Sci Rep.

[37] Pombero A, Bueno C, Saglietti L, Rodenas M, Guimera J, Bulfone A, Martinez S (2011). Pallial origin of basal forebrain cholinergic neurons in the nucleus basalis of meynert and horizontal limb of the diagonal band nucleus. Development.

[38] Proudman RGW, Akinaga J, Baker JG. The signaling and selectivity of α-adrenoceptor agonists for the human α2A, α2B and α2C-adrenoceptors and comparison with human α1 and β-adrenoceptors. *Pharmacol Res Perspect* e01003(2022). 10.1002/prp2.1003 PMC9471048 36101495

[39] Harris NA, Isaac AT, Günther A, Merkel K, Melchior J, Xu M, Eguakun E, Perez R, Nabit BP, Flavin S, Gilsbach R, Shonesy B, Hein L, Abel T, Baumann A, Matthews R, Centanni SW, Winder DG (2018). Dorsal BNST α2A-Adrenergic receptors produce HCN-Dependent excitatory actions that initiate anxiogenic behaviors. J Neurosci.

[40] Knapp JA, Morris NP, Henderson Z, Matthews RT (2000). Electrophysiological characteristics of non-bursting, glutamate decarboxylase messenger RNA-positive neurons of the medial septum/diagonal band nuclei of guinea-pig and rat. Neuroscience.

[41] Morris NP, Harris SJ, Henderson Z (1999). Parvalbumin-immunoreactive, fast-spiking neurons in the medial septum/diagonal band complex of the rat: intracellular recordings in vitro. Neuroscience.

[42] Damborsky JC, Smith KG, Jensen P, Yakel JL (2017). Local cholinergic-GABAergic circuitry within the basal forebrain is modulated by galanin. Brain Struct Funct.

[43] Damborsky JC, Yakel JL (2021). Regulation of hippocamposeptal input within the medial septum/diagonal band of Broca. Neuropharmacology.

[44] Hermanstyne TO, Kihira Y, Misono K, Deitchler A, Yanagawa Y, Misonou H (2021). Immunolocalization of the voltage-gated potassium channel Kv2.2 in GABAergic neurons in the basal forebrain of rats and mice. J Comp Neurol.

[45] Kiss J, Patel AJ, Baimbridge KG, Freund TF (2021). Topographical localization of neurons containing parvalbumin and choline acetyltransferase in the medial septum-diagonal band region of the rat. Neuroscience.

